# Bilateral Maxillary Fourth and Fifth Molars in a 17-Year-Old Female

**DOI:** 10.7759/cureus.115718

**Published:** 2026-09-03

**Authors:** Hikari Wakao, Takahiro Takekawa, Karen Gomi, Akinori Moroi, Koichiro Ueki

**Affiliations:** 1 Department of Oral and Maxillofacial Surgery, Division of Medicine, Interdisciplinary Graduate School, University of Yamanashi, Chuo-shi, JPN; 2 Oral and Maxillofacial Surgery, Fujiyoshida Municipal Hospital, Fujiyoshida, JPN

**Keywords:** fifth molar, fourth molar, supernumerary tooth, third molar, tooth extraction

## Abstract

Supernumerary teeth are relatively common in clinical practice; however, the occurrence of multiple supernumerary teeth in a bilaterally symmetrical pattern is rare. A 17-year-old female was referred to our department for extraction of impacted third molars. Panoramic radiography revealed two bilaterally symmetrical tooth-like structures adjacent to each maxillary third molar. Computed tomography confirmed that these were independent structures, leading to a diagnosis of bilateral maxillary fourth and fifth molars. In July 2023, the bilateral maxillary and mandibular third molars, together with the bilateral maxillary fourth and fifth molars, were extracted under general anesthesia. Gross examination of the extracted teeth revealed morphological features consistent with molar teeth. This case highlights the importance of accurate three-dimensional imaging and individualized treatment strategies in the management of complex and rare presentations of supernumerary teeth.

## Introduction

The reported prevalence of supernumerary teeth (ST) ranges from 0.1% to 3.8%, and most cases remain asymptomatic [1-5]. ST are often incidentally detected during radiographic examinations performed for dental treatment, such as caries, periodontal disease, or malocclusion [6-8]. Two-dimensional (2D) radiographic imaging has limitations in the diagnosis of supernumerary teeth, whereas three-dimensional (3D) evaluation using computed tomography (CT) is essential for accurate localization and morphological assessment.

Previous reports have suggested that ST are associated with genetic factors and systemic conditions, including syndromes [1]. ST may cause various clinical complications, such as delayed eruption, displacement of adjacent teeth, crowding, and cyst formation. The most common site of occurrence is the maxillary midline region, followed by the maxillary molar region [9-14].

Among supernumerary molars, fourth molars that morphologically resemble adjacent molars have occasionally been reported, whereas fifth molars are extremely rare.

## Case presentation

In April 2023, a 17-year-old female was referred to our department for extraction of impacted third molars. The patient had initially visited a local dental clinic, where impacted third molars were identified during a routine radiographic examination. Extraoral examination revealed a symmetrical facial appearance (Figure 1).

**Figure 1:**
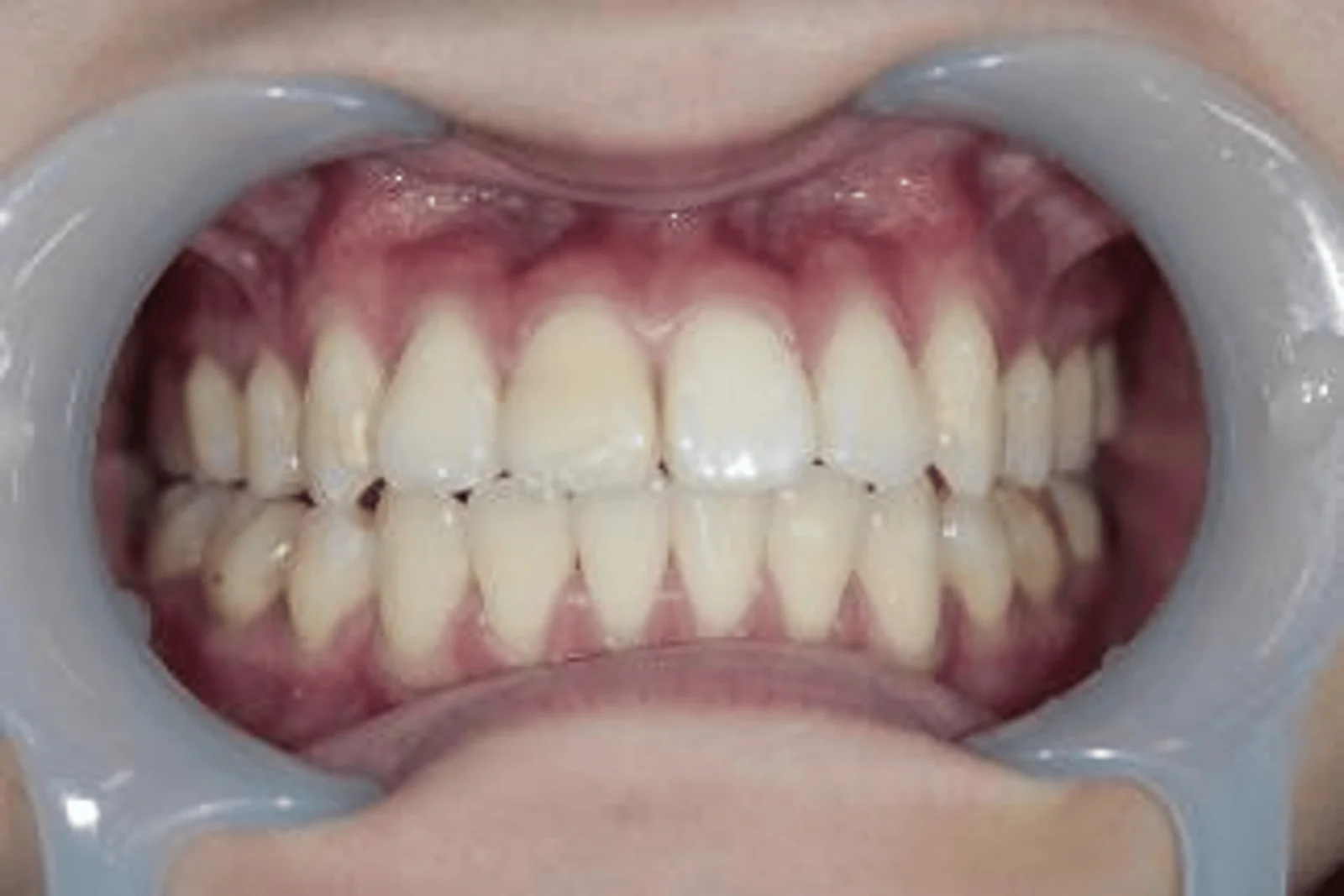
Intraoral photograph at the initial visit. Intraoral photograph showing that the dental midline was coincident on occlusion.

Intraoral examination showed that the dental midline was coincident and that the bilateral maxillary third molars remained unerupted distal to the second molars (Figure 2).

**Figure 2:**
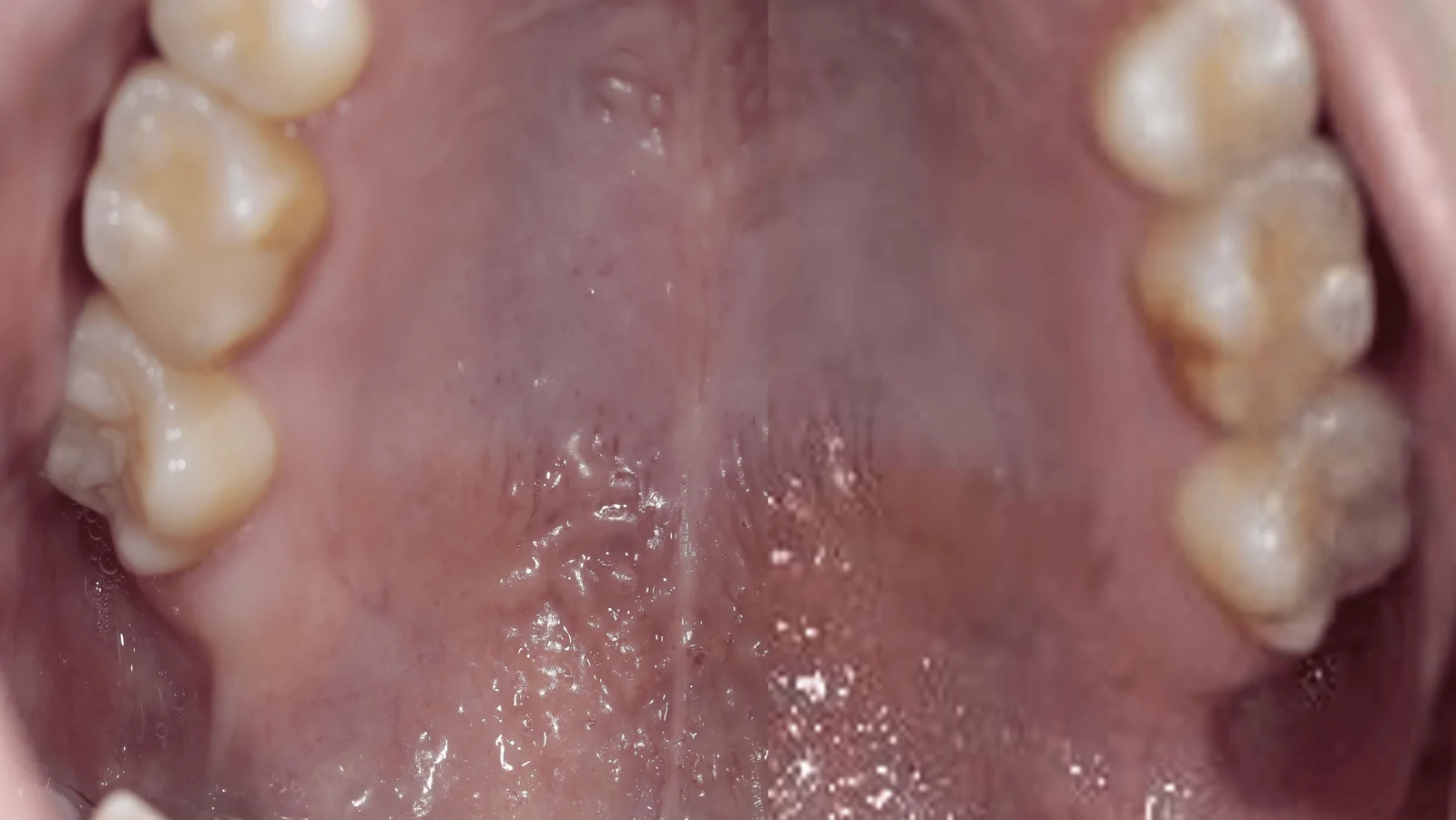
Intraoral photograph at the initial visit. Intraoral photograph showing that the bilateral maxillary third molars were unerupted.

The patient was of medium build and in good general health.

Radiographic and imaging findings

Panoramic radiography revealed complete impaction of all four third molars. In addition, two bilaterally symmetrical tooth-like radiopaque structures were observed adjacent to each maxillary third molar (Figure 3).

**Figure 3:**
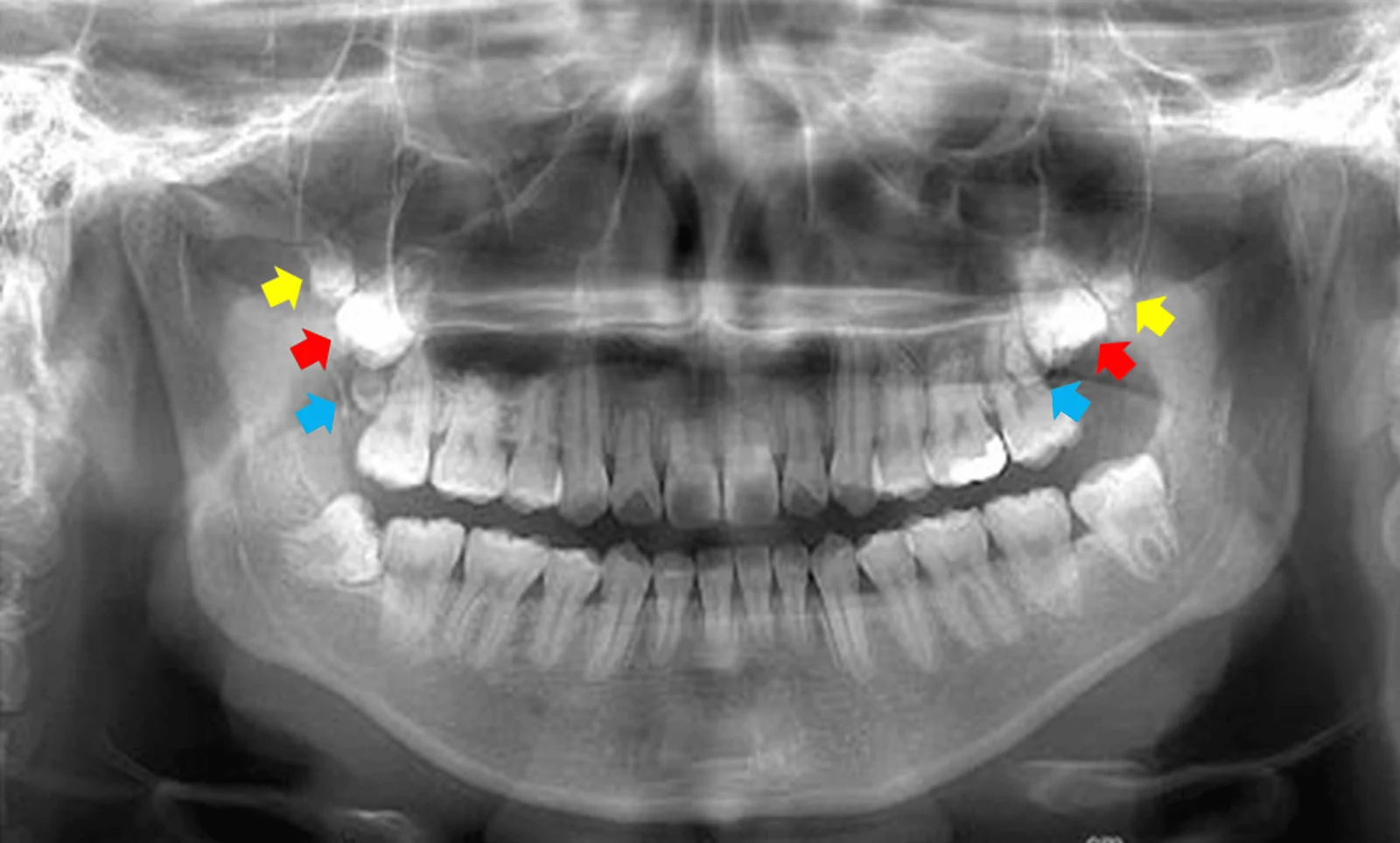
Panoramic radiograph at the initial visit. Two radiopaque tooth-like structures were identified around each maxillary third molar bilaterally. The red arrows indicate the maxillary third molars, the blue arrows indicate the maxillary fourth molars, and the yellow arrows indicate the maxillary fifth molars.

To further evaluate these findings, computed tomography (CT) with 3D reconstruction was performed. The CT images demonstrated two independent supernumerary teeth located distal to each maxillary third molar (Figures 4, 5).

**Figure 4:**
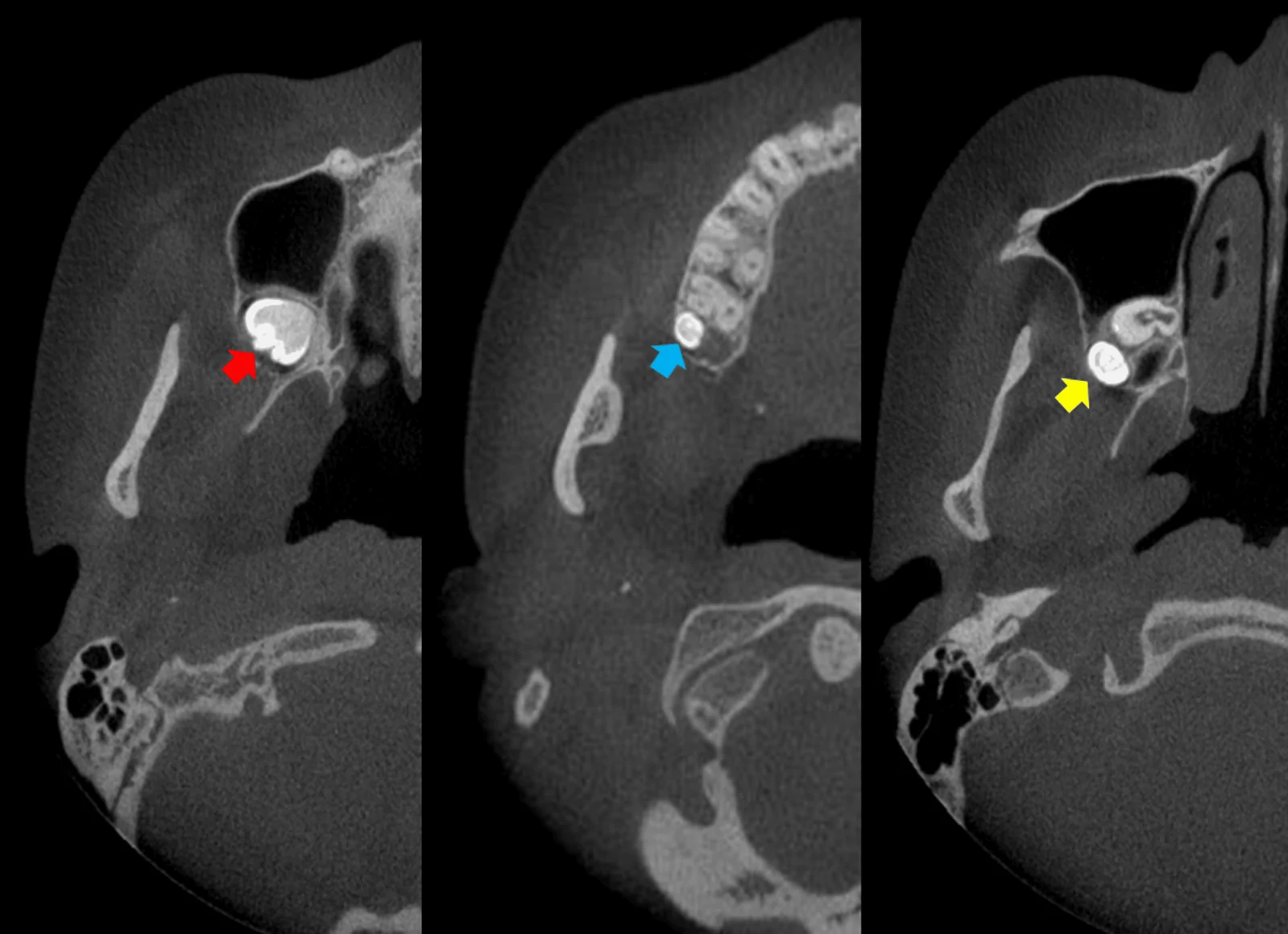
Axial computed tomography image at the initial visit. The right maxillary third molar (red arrow), fourth molar (blue arrow), and fifth molar (yellow arrow) were impacted.

**Figure 5:**
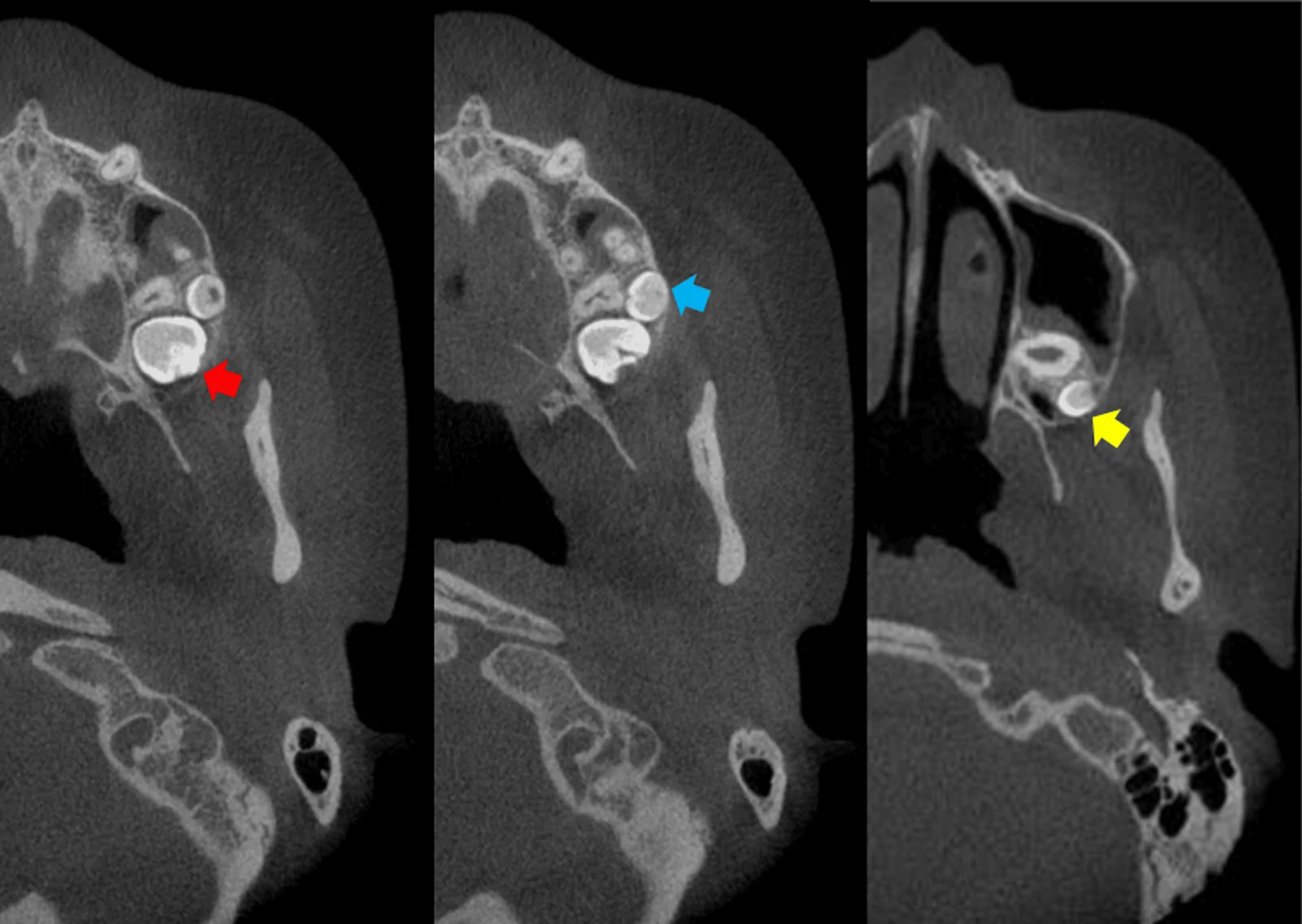
Axial computed tomography image at the initial visit. The left maxillary third molar (red arrow), fourth molar (blue arrow), and fifth molar (yellow arrow) were impacted.

Three-dimensional reconstructed images further confirmed their morphology and spatial relationship with the impacted third molars (Figures 6A, 6B).

**Figure 6:**
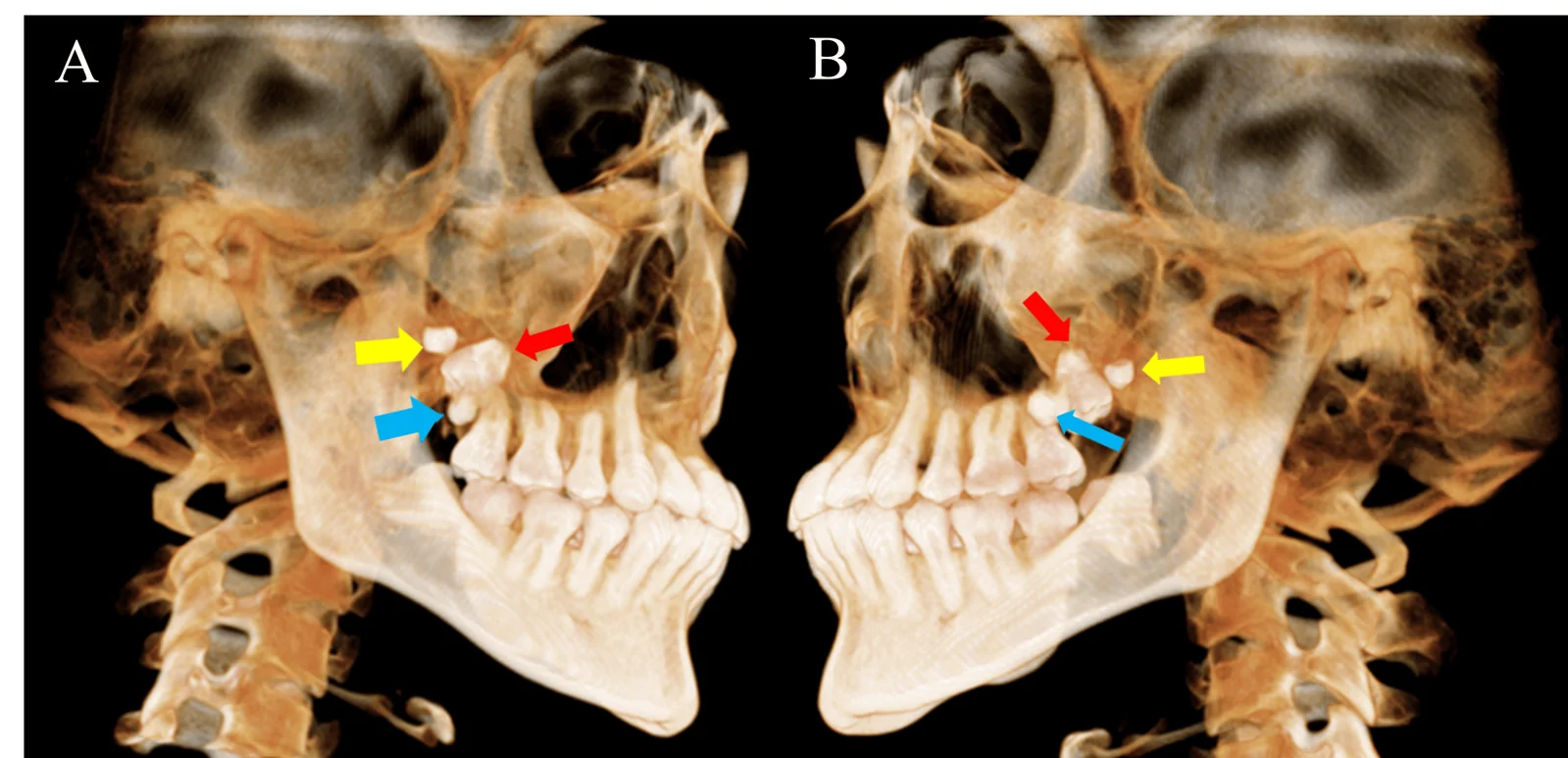
Three-dimensional reconstructed computed tomography images. A-B: One tooth-like structure was observed on the buccal side and another on the distal side of each bilateral maxillary third molar, each presenting as an independent tooth-like morphology. The maxillary third (red arrow), fourth (blue arrow), and fifth (yellow arrow) molars are indicated.

Based on the radiographic and CT findings, a diagnosis of bilateral maxillary fourth and fifth molars associated with impacted third molars was established.

Surgical management

In July 2023, the bilateral mandibular third molars and the bilateral maxillary third, fourth, and fifth molars were extracted under general anesthesia. Gross examination of the four maxillary supernumerary molars revealed morphological features consistent with molar teeth (Figure 7).

**Figure 7:**
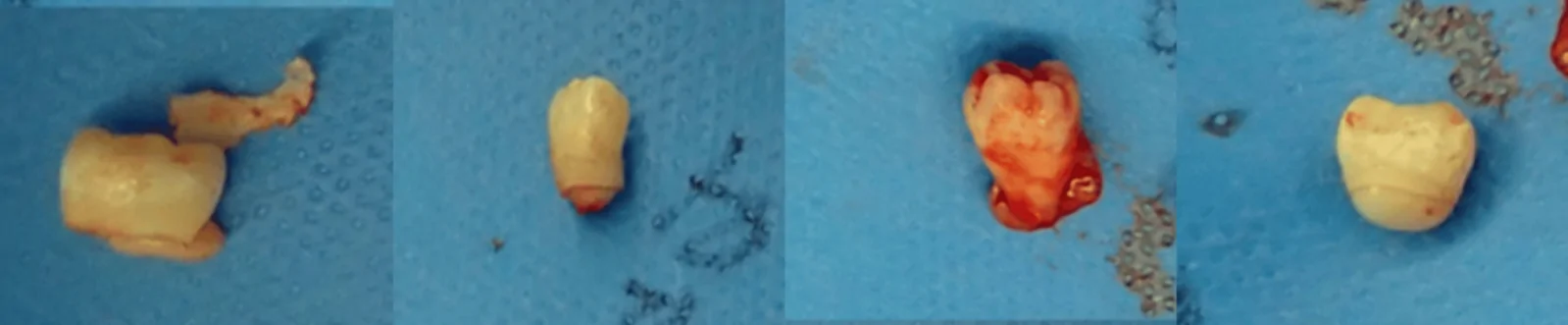
Photograph of the extracted specimens. From left to right, the extracted specimens include the right maxillary fifth molar, the right maxillary fourth molar, the left maxillary fourth molar, and the left maxillary fifth molar.

Postoperative outcome

Intraoperative findings revealed no oroantral communication between the extraction sockets and the maxillary sinuses. Each extracted tooth was independent, with no evidence of synodontia or adhesion to the surrounding alveolar bone or adjacent teeth. Gross examination of the extracted teeth revealed morphological features consistent with molar teeth.

The postoperative course was uneventful. Postoperative panoramic radiography demonstrated satisfactory healing of the extraction sockets, with no retained fragments or other abnormalities (Figure 8).

**Figure 8:**
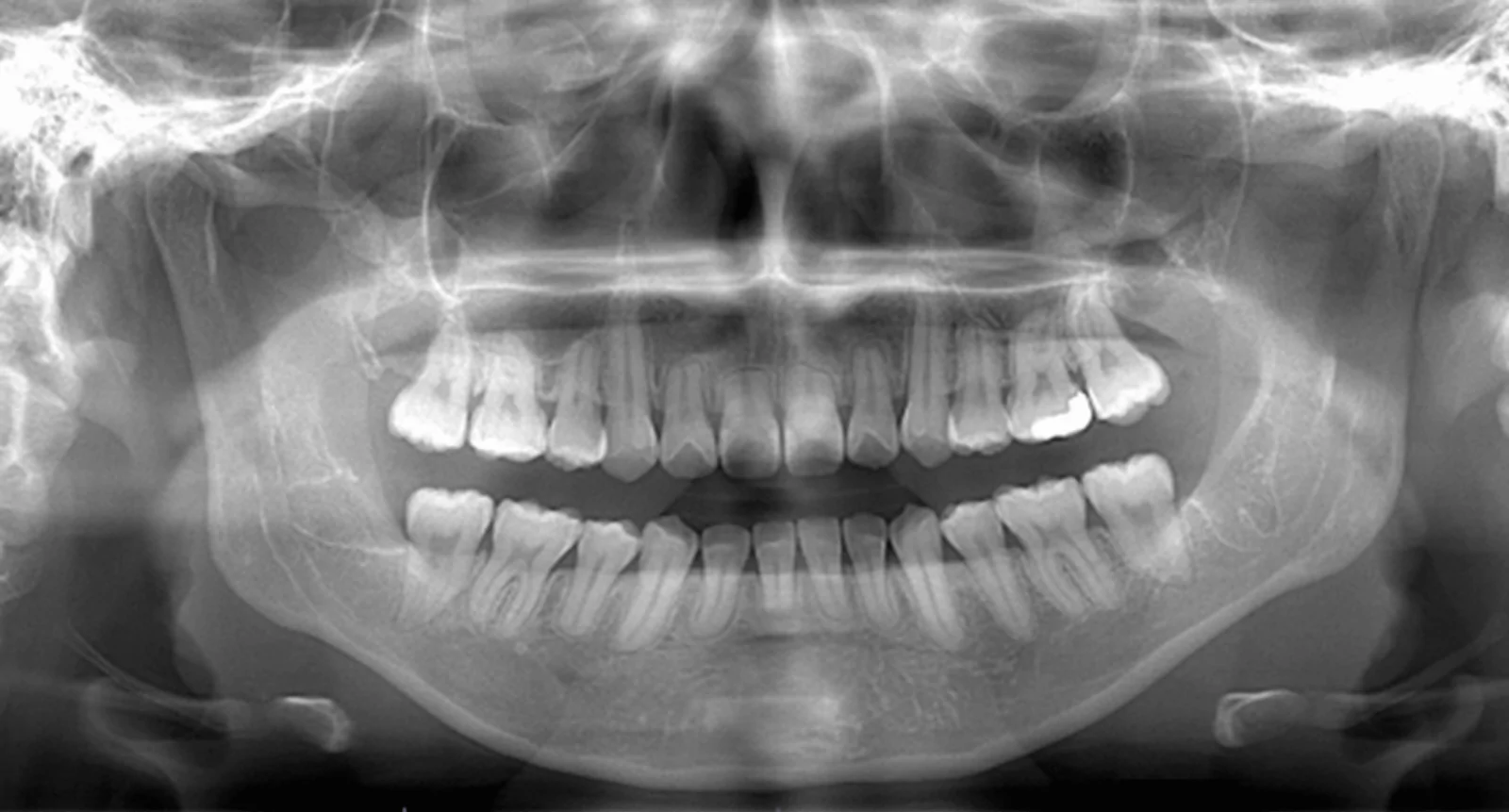
Postoperative panoramic radiograph. No abnormalities were observed at the extraction sites. The bilateral maxillary third, fourth, and fifth molars and the bilateral mandibular third molars had been completely removed.

The patient was followed for one year after surgery. At the one-year follow-up, clinical examination showed satisfactory healing, with no postoperative complications.

Written informed consent for the publication of this case and the accompanying images was obtained from the patient’s legal guardian, and assent was obtained from the patient.

## Discussion

The occurrence of ST is believed to be influenced by genetic and environmental factors. These teeth are more prevalent in the permanent dentition than in the primary dentition and are observed more often in the maxilla than in the mandible [1-5]. Regarding site-specific distribution, reported frequencies are as follows: maxillary anterior region, 77.2%; maxillary molar region, 11.0%; mandibular premolar region, 5.2%; mandibular anterior region, 2.7%; maxillary premolar region, 2.6%; and mandibular molar region, 2.7% [9]. These variations in frequency across different sites have been attributed to several factors, including the theory of phylogenetic atavism and spatial variations within the successional dental lamina [9,12,13].

In a survey of children aged 5-15 years with ST, 4.6% had three or more supernumerary teeth [1]. Furthermore, five or more supernumerary teeth were observed in only 2.4% (6 of 251) of affected individuals [7]. Fujiwara et al. revealed that among patients with bilaterally symmetrical ST, the sites of occurrence were the anterior (73.3%; 33 cases), premolar (8.9%; 4 cases), and molar (8.9%; 4 cases) regions [8]. In the present case, four ST were observed in the bilateral maxillary third molar region, with two teeth occurring symmetrically on each side, a rare clinical presentation based on the number and the specific posterior location.

Systemic conditions associated with a high prevalence of ST include cleft lip and palate and cleidocranial dysplasia. Other reported associations include Gardner syndrome, chondroectodermal dysplasia (Ellis-Van Creveld syndrome), Ehlers-Danlos syndrome, and tricho-rhino-phalangeal syndrome [1]. CT is an essential diagnostic tool for ST, providing accurate 3D information regarding tooth orientation, local abnormalities, and relationships with adjacent anatomical structures. In this case, 3D CT reconstruction aided in reducing operative time and surgical invasiveness.

Compound and complex odontomas were considered in the differential diagnosis. However, each radiopaque structure appeared as an independent tooth-like structure with a recognizable crown and root morphology resembling that of a molar. These findings differed from the multiple small tooth-like structures typically observed in compound odontomas and the irregular radiopaque mass characteristic of complex odontomas. Therefore, based on the panoramic radiographic, CT, and gross morphological findings, the structures were diagnosed as bilateral maxillary fourth and fifth molars. However, histopathological examination of the extracted specimens was not performed; therefore, their dental tissue composition could not be microscopically confirmed, and odontomas could not be definitively excluded. This represents a limitation of the present case.

To our knowledge, only 10 cases of bilateral maxillary fourth molars, including the present case, have been reported in the literature [15-23] (Table 1).

**Table 1:** Reported cases of bilateral maxillary fourth molars.

Author, year [reference]	Country	Age (years)	Sex	Supernumerary teeth other than fourth molars	Treatment	Morphology
Wakumoto, 1956 [15]	Japan	26	Male	One right maxillary fifth molar	Extraction of all teeth except the right maxillary fifth molar	Atypical
Koyama et al., 2011 [16]	Japan	24	Female	Two bilateral maxillary fifth molars	Extraction	Atypical
Cavalcanti et al., 2011 [17]	Brazil	22	Male	None	Extraction	Atypical
Clementini et al., 2013 [18]	Italy	22	Male	None	Extraction	Atypical
Asrani et al., 2016 [19]	India	33	Female	None	Observation	Typical
Jaiyeoba et al., 2018 [20]	Nigeria	15	Male	None	Extraction	Atypical
Anisuzzaman et al., 2019 [21]	Bangladesh	27	Female	None	Extraction	Atypical
Al-Haj Husain et al., 2022 [22]	Switzerland	29	Female	None	Extraction	Typical
Takeo et al., 2024 [23]	Japan	17	Male	One supernumerary tooth in the maxillary anterior region	Extraction	Typical and atypical
Present case	Japan	17	Female	Bilateral maxillary fifth molars	Extraction	Typical

These studies suggest that, in addition to genetic factors, racial and geographic factors may influence ST occurrence. Reportedly, variations exist in prevalence among racial groups [1,2,5,8,14], with a higher prevalence reported in Black populations (6.4%) than in White populations (0.9%). Similarly, other studies have indicated a higher prevalence in Asian populations (2.7%) than in White populations (1.6%). Notably, 4 of the 10 reported cases were from Japan. This relatively high frequency of reported fourth and fifth molars in East Asia, particularly Japan, suggests potential regional variation in their manifestation.

The presence of ST may cause malocclusion, including tooth displacement, crowding, delayed eruption, or impaction, as well as root resorption. Therefore, extraction is the preferred treatment when an adverse impact on adjacent teeth is expected [3,6]. Clinical factors associated with these complications include morphology, orientation, and the patient’s age [24]. Currently, there is no consensus on the optimal timing for ST extraction. The existing literature highlights two primary approaches: early intervention before root formation of permanent teeth and delayed extraction after root formation is complete [1,25].

Additionally, in cases where simultaneous extraction of the third and fourth molars is deemed high-risk, a staged approach has been proposed. This approach involves prioritizing the extraction of the third molar, followed by a period of clinical observation until the fourth molar erupts, at which point it is subsequently removed [26]. However, in some cases, extraction may be challenging, such as when the ST is fused to an adjacent tooth at the crown or root, or when it is located close to the root apex of an adjacent tooth or the nasal floor. In such cases, if permanent tooth eruption is favorable and no associated cystic or tumorous lesions are present, careful observation may be considered a treatment option. During follow-up, periodic radiographic examinations are required to confirm the position of the ST and to monitor for the development of cystic or tumorous lesions [27-29]. In this case, extraction was indicated based on a comprehensive preoperative assessment, in which we considered the potential for malocclusion in the permanent dentition and the risk that the ST could become a source of inflammation. Surgical planning using 3D CT enabled a minimally invasive extraction process, underscoring that the management of ST necessitates accurate 3D diagnosis and a tailored treatment strategy for each patient.

## Conclusions

ST are influenced by genetic and environmental factors and are more commonly associated with permanent dentition and the maxilla. The present case, which involved four bilaterally symmetrical ST in the maxillary third molar region, represents a rare clinical presentation regarding the number of teeth and their posterior anatomical location. Accurate diagnosis using 3D CT was essential for a precise evaluation of anatomical relationships and facilitated a minimally invasive surgical approach. ST management should be individualized, considering tooth morphology, orientation, potential complications, and patient-specific factors. This case highlights the importance of accurate 3D imaging and tailored treatment strategies in the management of complex and uncommon presentations of ST.
